# Internal Fixation of Only the Distal End in a Bipolar Segmental Clavicle Fracture: A Case Report

**DOI:** 10.5704/MOJ.1711.003

**Published:** 2017-11

**Authors:** T Ogawa, T Sasaki, MK Masayuki-Kawashima, A Okawa, MK Mahito-Kawashima

**Affiliations:** Department of Orthopaedics, Tokyo Medical and Dental University, Tokyo, Japan; ^*^Department of Orthopaedics, Kawashima Orthopedic Hospital, Oita, Japan

**Keywords:** bipolar segmental clavicle fracture, surgical fixation, unilateral locking plate fixation, distal-only fixation

## Abstract

Bipolar segmental clavicle fractures are simultaneous clavicle fractures of both proximal and distal ends. Few case reports describing these fractures have been published, and the management of these injuries have remained controversial. Non-operative treatment is likely to result in poor shoulder function due to the instability of the fracture in patients with high physical demands. In contrast, surgical treatment with fixation of both proximal and distal ends of the clavicle possibly may cause life-threatening complications. We present a 74-year old female farmer who had injured her left shoulder and was diagnosed with a bipolar segmental clavicle fracture. Taking the fracture mechanism into consideration, we surgically treated only the distal end of the clavicle fracture with a locking plate. The proximal end of the clavicle fracture was treated without surgical intervention. Both fracture sites achieved bony union after four months and she returned to her activities as a farmer. Quick DASH score was 5.0 with excellent results at three years after operation.

## Introduction

Clavicle fractures are common and most occur in the mid-shaft of the clavicle. Simultaneous fractures of the proximal and distal end of the clavicle, however, are rare and known as bipolar segmental clavicle fractures^1^. Management of bipolar segmental clavicle fractures has been controversial, ranging from non-operative to surgical treatments. For surgery, most authors have reported fixation as a treatment option for both proximal and distal ends of the clavicle fractures due to fracture instability^1,2^. However, the fixations of the proximal clavicle fracture may cause severe complications^3^. In this article, we report a case of a left bipolar segmental clavicle fracture in an elderly female who underwent surgical treatment. Surgery was performed only for the distal end of the clavicle alone, while the proximal end was treated nonoperatively.

## Case Report

**Fig. 1:**
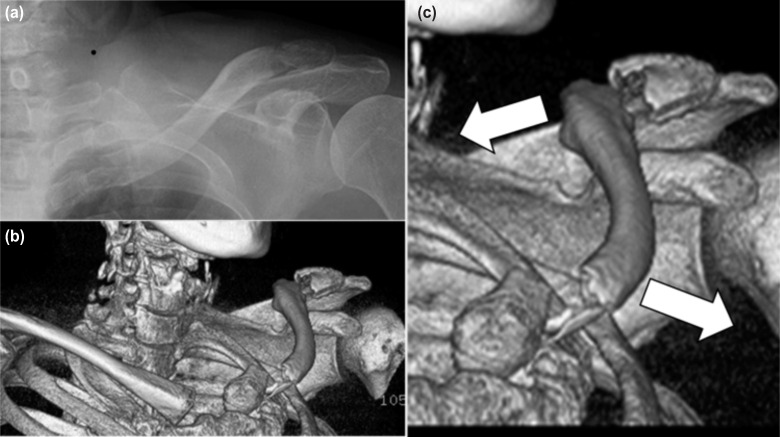
(a) Preoperative standard radiograph, (b) Preoperative three-dimensional CT scan image, (c) Proximal end of the clavicle was displaced anteriorly and the distal end posteriorly.

A 74-year old female farmer had injured her shoulder against the side view mirror of a moving car. After the accident, she was unable to lift her left arm due to shoulder pain and attended our hospital. On physical examination, a deformity was observed on the left clavicle. Standard radiograph revealed a fracture at the distal end of the left clavicle (Fig. 1a). Three-dimensional computer tomography (CT) scan revealed in addition a displaced fracture of the proximal end of the clavicle, and a diagnosis of bipolar segmental clavicle fracture was made (Fig. 1b). The proximal and distal displaced fractures were extra-articular (Robinson Type 1B1) and intra-articular (Robinson Type 3B2), respectively. The proximal end of the clavicle was displaced anteriorly and the distal end posteriorly (Fig. 1c), with floating intermediate fracture fragments. This indicated fracture instability and a high risk of non-union^1^. Therefore, we decided to treat with open reduction, and it was performed seven days after injury with the patient’s consent.

The patient underwent surgery under general anaesthesia in the beach chair position. A skin incision was made over the distal fracture site. The distal end of the clavicle was displaced posteriorly. The floating bone fragment was found rotated opposite to the direction of the displacement. As the distal end of the clavicle was reduced, the proximal end was simultaneously reduced to a good anatomical position. A 3-hole locking plate [LCP™ Superior Clavicle Plate, DePuy Synthes Trauma, West Chester, USA] was used to provide fixation between the distal end of the clavicle, as the intermediate floating fracture fragment was secured by more than two screws to prevent rotational displacement. The proximal clavicle fracture was left alone to avoid any critical complications.

**Fig. 2:**
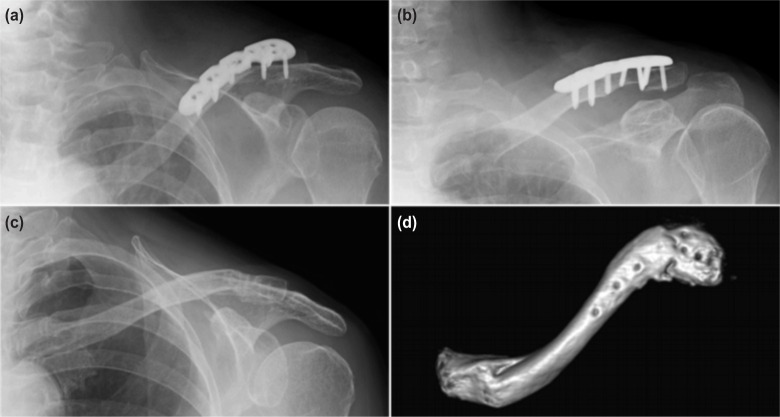
(a,b) Standard radiographs at four months after operation, (c) and after implant removal at 14 months after operation, (d) Three-dimensional CT scan image after implant removal.

**Fig. 3:**
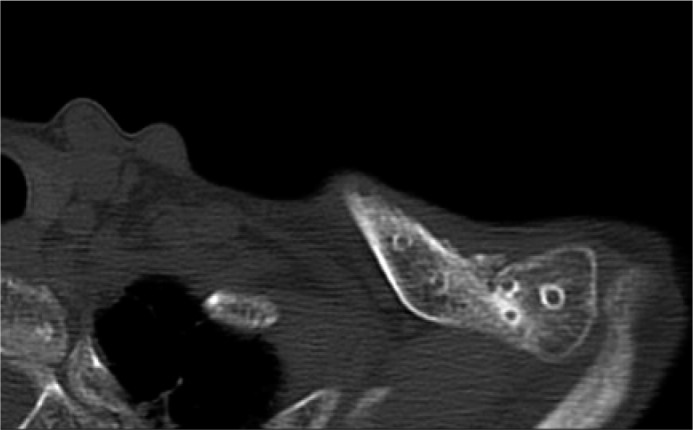
CT scan image after implant removal at 14 months after operation. Bony union was achieved.

After the operation, the patient’s shoulder was placed in a sling and allowed passive range of motion of the shoulder below 90 degrees for one month as the proximal clavicle fracture was not surgically fixed. Gradually active movement of the shoulder was allowed as tolerated. Four months after the operation, the patient was able to elevate her shoulder forward up to 160 degrees. Radiographs and CT scans revealed bony union of both proximal and distal ends of the fracture (Fig. 2a and b). Because of discomfort due to implant irritation, the implant was removed one year after surgery and the symptoms diminished (Fig. 2c, d and Fig. 3). Three years post operatively, the patient had neither pain nor crepitation in both her proximal and distal fracture sites. The ROM of shoulder was 165 degrees in anterior elevation and 80 degrees in abduction. The Oxford shoulder score was 47 points and ASES shoulder score was 91.6 points. The Quick DASH score was 5.0 points, demonstrating a better score than previous literatures (8 points without complication case, 66 points with complication case)^3^. The patient has continued her activities as a farmer.

## Discussion

Bipolar segmental clavicular fracture was first described by Porral in 1831^4^. There is still no consensus regarding the treatment of bipolar segmental clavicle fractures. Previous reports have suggested that bipolar segmental clavicle fractures usually occur as a result of high energy trauma. However, low energy trauma such as falling on the outstretched hand can also cause this injury^1^.

Non-operative treatment is recommended for minimally displaced fractures in low-demand patients^5^. However, some studies report gross deformity of the clavicle and poor functional outcome following non-surgical treatment. Even if the fracture is caused by low-energy trauma, the instability of the shoulder can persist; thus, operative treatment is recommended^1^. According to previous reports, most surgical management includes fixation for both proximal and distal ends of the clavicle^1,3^. With regard to surgical treatment, proximal clavicle fractures lead to serious complications during operation, such as screw penetration into the pericardium and pneumothorax^3^.

The injury mechanisms of segmental clavicle fractures have been unknown. In our case, a plausible explanation of the injury mechanism is that the patient first hit her shoulder from the anterior to posterior direction that forced her upper limb to displace in excessive stretch, inducing a rotational force on her clavicle. Therefore, the sternoclavicular and coracoclavicular joints acted as fulcrum and resulted in simultaneous fractures of the proximal and distal ends of the clavicle.

In consideration of the above injury mechanism, at operation we rotated the patient’s intermediate clavicle fragment towards the direction of displacement and provided fixation by a locking plate. As a result, the angular stability of the locking plate stabilized the fragment in the anatomical position and dispersed the motion stress of both fracture sites. Because our treatment did not provide rigid fixation, the patient’s shoulder was immobilized after operation. Although early mobilization is possible with surgical intervention, we believe the disadvantage of postoperative immobilization does not outweigh the fatal complications that may follow surgical fixation of the proximal clavicle fracture. Ultimately, it was gratifying that both fracture sites achieved bony union and acceptable shoulder function.

In conclusion, unilateral locking plate fixation of the distal end can be a useful option for treating bipolar segmental clavicle fractures.
